# Correlation of DCE-MRI Perfusion Parameters and Molecular Biology of Breast Infiltrating Ductal Carcinoma

**DOI:** 10.3389/fonc.2021.561735

**Published:** 2021-10-13

**Authors:** Li Liu, Nan Mei, Bo Yin, Weijun Peng

**Affiliations:** ^1^ Department of Radiology, Fudan University Shanghai Cancer Center, Shanghai, China; ^2^ Department of Oncology, Fudan University Shanghai Cancer Center, Shanghai, China; ^3^ Department of Radiology, Huashan Hospital, Fudan University, Shanghai, China

**Keywords:** dynamic contrast-enhanced magnetic resonance imaging (DCE-MRI), breast tumor, biological molecule, Ki-67, infiltrating ductal carcinoma

## Abstract

**Objective:**

We aimed to investigate the correlation of the perfusion parameters of dynamic contrast-enhanced magnetic resonance imaging (DCE-MRI) with the molecular biological expression of breast infiltrating ductal carcinoma (IDC) in order to guide appropriate therapeutic advice and clinical outcome prediction.

**Materials and Methods:**

In a prospective analysis of 67 patients with breast IDC, preoperative DCE-MRI and routine MRI images were obtained. The double-chamber model (extended Tofts model) was employed to calculate the perfusion parameters. Postoperative pathological immunohistochemistry was examined, including human epidermal growth factor receptor 2 (HER-2), estrogen receptor (ER), progesterone receptor (PR), cell nuclear-associated antigen (Ki-67), cytokeratin 5/6 (CK5/6), and epidermal growth factor receptor (EGFR). Statistical analysis was applied to explore the relationship between the perfusion parameters and the molecular biomarkers of breast cancer.

**Results:**

A total of 67 lesions were included in our study. The mean maximum diameter of lesions was 4.48 ± 1.73 cm. Perfusion parameters had no correlation with tumor diameters (*p* > 0.05). The volume transfer constant (*K* ^trans^) and the rate constant (*k*_ep_) had positive correlations with Ki-67 (*p* < 0.05). The plasma volume ratio (*v*_p_) had a statistical difference between CK5/6 positivity and CK5/6 negativity. The maximum rising slope (MAX Slope) was higher in HER-2-enriched tumors than that in luminal A or B tumors (*p* < 0.05). *k*_ep_ was higher in HER-2-enriched tumors than that in luminal A tumors (*p* < 0.05). The extravascular extracellular space volume fraction (*v*_e_) was higher in triple-negative tumors than that in HER-2-enriched and in luminal A and B tumors (*p* < 0.05). The time to peak enhancement (TTP) was lower in HER-2-enriched tumors than that in luminal A and B tumors (*p* < 0.05). Maximum concentration (MAX Conc) was higher in triple-negative tumors than that in luminal B tumors (*p* < 0.05).

**Conclusion:**

DCE-MRI perfusion parameters can behave as a noninvasive tool to assess the molecular biological expression and the molecular subtypes of breast IDC. They may aid in predicting breast IDC invasiveness, metastasis, and prognosis.

## Introduction

Breast cancer is the most commonly diagnosed malignancy among women, with an incidence rate of 268.6 per 100,000 and a mortality rate of 69.5 per 100,000 in 2015. Breast infiltrating ductal carcinoma (IDC) is the most common type of breast cancer (1).

In clinical practice, there are many widely used methods to diagnose breast cancer, such as palpation, mammography (MM), ultrasonography (US), and magnetic resonance imaging (MRI). With the clinical application of high field intensity MRI and the dedicated breast coil, the sensitivity of breast MRI diagnosis is as high as 88%–100% (2). The conventional breast dynamic contrast-enhanced magnetic resonance imaging (DCE-MRI) is widely employed in clinical practice, which distinguishes malignant tumors of the breast from benign tumors on the basis of the blood flow dynamic curve and calculates the semiquantitative blood flow perfusion parameters. Nevertheless, based on appropriate pharmacokinetic models, DCE-MRI carries the ability to calculate quantitative perfusion parameters, which is of significant value in differentiating malignant lesions from benign lesions and in assessing the response of breast tumors to neoadjuvant chemotherapy (NAC) (3).

Breast IDC with different expression levels of Ki-67, human epidermal growth factor receptor 2 (HER-2), estrogen receptor (ER), and progesterone receptor (PR) may result in remarkably different prognoses, and distinct molecular subtypes need different treatment strategies (4). Hence, evaluating the molecular biological features of breast IDC noninvasively before surgery plays a significant role in guiding personalized treatment.

DCE-MRI reflects the blood perfusion and is related to the biological characteristics of the tumor. Therefore, this technology has great potential in evaluating the molecular biological characteristics of the tumor. Some researchers have used perfusion-weighted images (PWIs) to evaluate the molecular biological characteristics of breast cancer, concluding that the parameters of PWIs are correlated with the molecular biological characteristics of breast cancer. However, the results varied among studies, which could be explained by the samples including different pathological types of breast cancer (5–7). Thus, this study focuses on IDC as it is the most common type.

The purpose of our study was to investigate the correlation between DCE-MRI perfusion parameters and the molecular biological characteristics of breast IDC at the aim of arriving at appropriate therapeutic advice and prognosis prediction.

## Materials and Methods

### Patients

All 102 patients, clinically diagnosed with suspicious breast cancer in Fudan University Shanghai Cancer Center between May 2018 and May 2019, underwent radical mastectomy or breast-conserving surgery. Patients were excluded if they were diagnosed with non-infiltrating ductal carcinoma; were after percutaneous biopsy, radiation therapy, or chemotherapy; were receiving hormone replacement therapy; or were lactating. Ultimately, 67 consecutive patients pathologically diagnosed with breast IDC after operation (1 male and 66 females, mean age ± standard deviation = 48.7 ± 9.7 years, range = 25–68 years) were enrolled in our study.

This study was approved and reviewed by the Institutional Review Board (IRB) and the requirement of informed patient consent was waived.

### Methods

#### Breast MRI Examination Protocol

The MRI scan time of female patients was selected in the second to the third week of the menstrual cycle, and the time interval between the MRI scan and the surgery was 1–14 days. All breast MR examinations were performed in a unified protocol using a 3-T system (GE SIGNA ExciteHDx3.0 T, Milwaukee, WI, USA) with the use of a dedicated breast coil (eight-channel phased array). Patients were asked to lie prone on the examination table with both breasts placed in the coil. To prevent patients from moving, the straps attached to the MR machine were used to fix the patients.

##### Routine Scanning

Conventional three-plane localizer images (coronary, sagittal, and transverse) were obtained, followed by the following sequences: 1) T1-weighted (T1W) fast spin echo series with a repetition time (TR)/echo time (TE) of 960/8.9 ms, a field of view (FOV) of 30 cm, 1 NEX, 4-mm slice thickness, no gap, and 352 × 192 matrix; 2) sagittal fat-suppressed T2-weighted (T2W) sequence using a TR/TE of 3,800/85 ms, an FOV of 20 cm, 2 NEX, 4-mm slice thickness, and no gap; 3) transverse T2W fat-suppressed images with a TR/TE of 5,220/48.2 ms, an FOV of 30 cm, 2 NEX, a slice thickness of 4 mm, no gap, and a matrix size of 352 × 192.

##### T1 Perfusion and Dynamic Scanning

Transverse 3D T1 gradient-echo scans were performed initially before contrast injection using three inversion angles of 5°, 10°, and 15° to obtain a map of the T1 baseline value (T1 map) and calculate the T1 value. Subsequently, gadolinium–diethylenetriaminepentaacetic acid (Magnevist) was injected into an antecubital vein using a high-pressure syringe at a dose of 0.2 mmol/kg of body weight and at a rate of 3 ml/s, followed by a 20-ml saline flush for all patients. After the contrast injection, dynamic contrast-enhanced scanning (pharmacokinetic perfusion scanning) was performed immediately through the entire breast using the same scan parameters as when capturing the baseline T1: volume imaging for breast assessment (VIBRANT) sequence cross-section scanning (TR/TE of 4.4/2.1 ms, layer thickness of 2 mm, no gap, matrix of 416 × 320, NEX 0.75 times, and FOV of 34 cm). A total of five phases were collected.

#### Calculation of Pharmacokinetic Perfusion Parameters

As a post-processing procedure, the DICOM files from DCE-MRI were transferred to a personal computer and processed to calculate the perfusion parameters using the Omni-Kinetics software (GE Healthcare, Chicago, IL, USA) and the double-chamber model (extended Tofts model). The following perfusion parameters were computed in accordance with the arterial input function (AIF): volume transfer constant (*K* ^trans^), extravascular extracellular space volume fraction (*v*_e_), rate constant (*k*_ep_), plasma volume ratio (*v*_p_), and the initial area under the concentration curve in 60 s (iAUC). Semi-quantitative parameters include the time to peak enhancement (TTP), the area under the concentration curve (AUC), maximum concentration (MAX Conc), and maximum rising slope (MAX Slope). These T1 perfusion parameters were all obtained by means of the double-chamber model, the population average AIF, and a linear least-squares fitting algorithm.

All acquired MR images were evaluated by two independent radiologists (BY and WP) with more than 5 years of experience with breast MRI diagnosis. Three imaging levels with the largest lesion including the area with the highest *K* ^trans^ were manually selected. The necrotic area for each lesion was discriminated out in reference to the T2W image and enhanced T1W image. The region of interest (ROI) involved the entire lesion and avoided the necrotic area as much as possible. The average of three imaging measurements were then calculated. Each lesion was also reconstructed in multiple planes to measure the maximum diameter.

#### Pathological Examination

All patients underwent immediate postoperative pathological examination: pathological type, tumor size, and immunohistochemical expressions of ER, PR, HER-2, and Ki-67. Forty-two patients were also tested for the expressions of cytokeratin 5/6 (CK5/6) and epidermal growth factor receptor (EGFR).

The status of ER, PR, and HER-2 was evaluated according to the standards recommended by the American Society of Clinical Oncology/College of American Pathologists (ASCO/CAP) (8). Antibodies used in the Ki-67 test and the buffer were purchased from Dako (Santa Clara, CA, USA). The clone number of the Ki67 immunohistochemistry antibody is MIB-1 (working concentration, 1:1,000). The EnVision secondary antibody detection kit was from EnVision Detection Systems (peroxidase/DAB, rabbit/mouse), and the primary antibody diluent was Antibody Diluent, which was made ready to use. The Ki-67 positivity percentages were calculated according to the percentage of positively stained tumor cells among the total number of cells assessed in the tumor.

Using mouse anti-human CK5/6 protein monoclonal antibody, the hematoxylin/eosin (HE)-stained section was observed under a microscope to determine the cancer nest, and the material was labeled to make a beeswax section. After the sections were deparaffinized and hydrated, they were then thermally repaired with a high-temperature antigen and finally stained. Cells with ≥5% CK5/6 expression were defined as positive and those with <5% were considered negative. The PV-9000 three-step immunohistochemical method was used to detect EGFR. EGFR was considered positive if a weak or strong positive staining was seen on the cytoplasm and membrane of tumor cells and the percentage of positive cells was above 10%.

According to the criterion set by the 12th St Gallen International Breast Cancer Conference (2011) (9), the various subtypes of breast cancer were defined in terms of immunohistochemistry.

### Statistical Analysis

All DCE-MRI perfusion parameters were expressed as the mean and standard deviation. The perfusion parameters in different molecular biological expression subtypes were compared using independent samples *t*-test. Comparisons of the perfusion parameters between different molecular subtypes of breast cancer and the corresponding distinct subgroups were analyzed using one-way analysis of variance (ANOVA) and least significant difference (LSD), respectively. Furthermore, Spearman’s rank correlation analysis was applied for the correlation of the perfusion parameters and the expression status of Ki-67, while Pearson’s correlation analysis was applied for the relevant analyses between tumor size and perfusion parameters. SPSS software (version 13.0) was employed for all statistical analyses. Statistical significance was assigned if the *p*-value was less than 0.05.

## Results

### Patient Information

Sixty-seven patients pathologically diagnosed with breast IDC after operation were enrolled in our study, with a total of 67 lesions.

### Correlation of DCE-MRI Perfusion Parameters and Tumor Size

In 67 cases, the mean tumor size was 4.48 ± 1.73 cm. It was illustrated by employing Pearson’s correlation analysis that tumor size was not significantly associated with any perfusion parameters (*p* > 0.05).

### Correlation of DCE-MRI Perfusion Parameters and Ki-67

**Figure 1:**
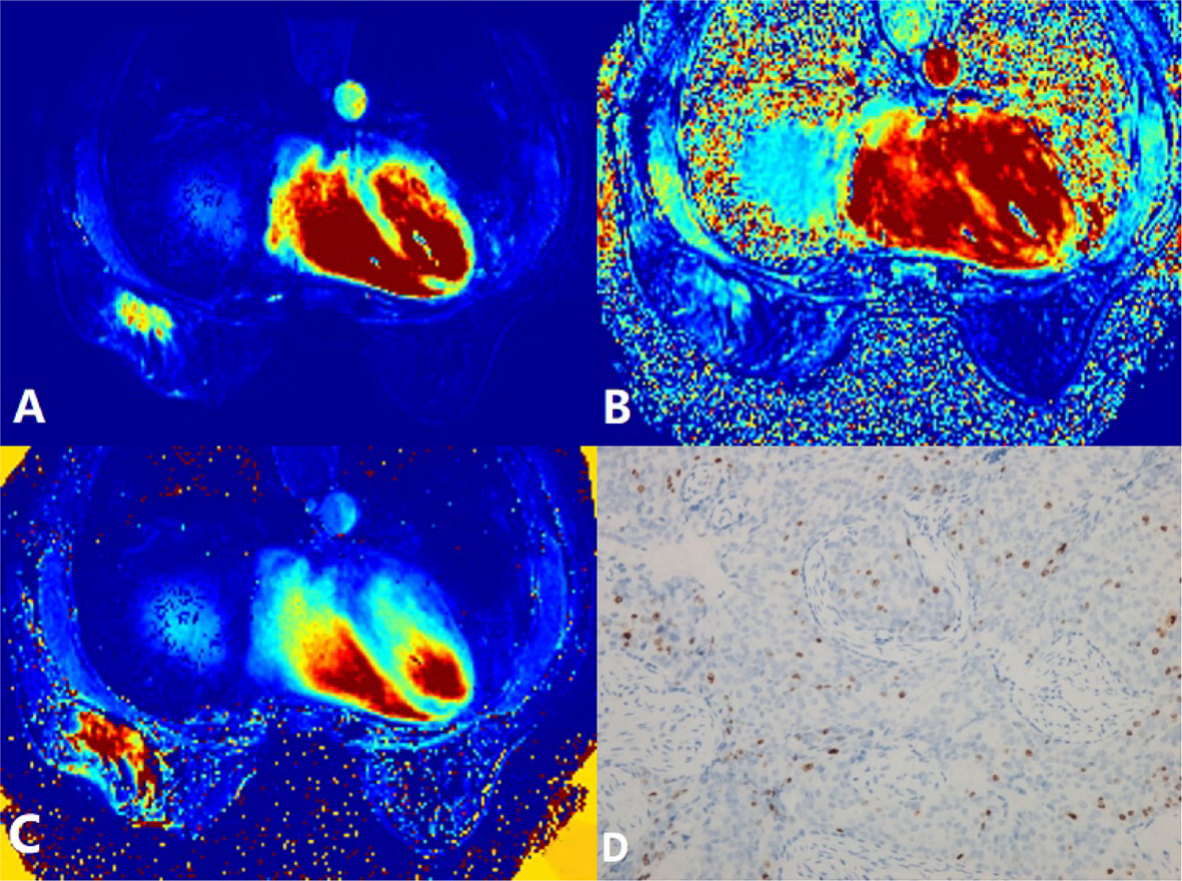
A 58-year-old woman postoperatively pathologically diagnosed with right breast infiltrating ductal carcinoma (IDC). **(A)** *K* ^trans^ = 0.28/min. **(B)** *k*_ep_ = 0.31/min. **(C)** *v*_e_ = 0.96. **(D)** Ki-67 positively stained (15%). *K^trans^*, volume transfer constant; *k*_ep_, rate constant; *v_e_*, extravascular extracellular space volume fraction.

**Figure 2:**
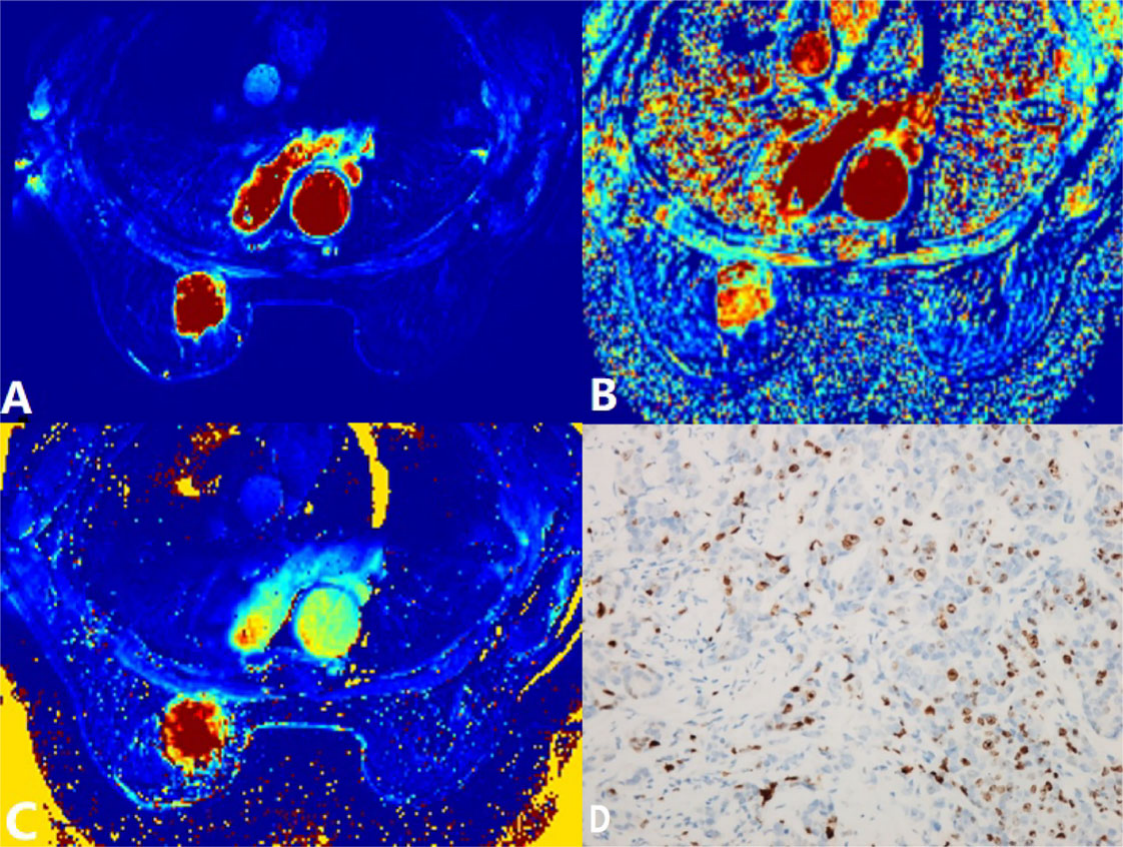
A 60-year-old woman postoperatively pathologically diagnosed with left breast infiltrating ductal carcinoma (IDC). **(A)** *K* ^trans^ = 0.76/min. **(B)** *k*_ep_ = 0.67/min. **(C)** *v*_e_ = 1.09. **(D)** Ki-67 positively stained (30%). *K^trans^*, volume transfer constant; *k*_ep_, rate constant; *v_e_*, extravascular extracellular space volume fraction.

**Table 1** shows the relationship between Ki-67 and the MRI perfusion parameters that were analyzed. It is worth noting that *K* ^trans^ and *k*_ep_ were positively correlated with the Ki-67 status of the lesions (*p* < 0.05). Borderline correlations were found between *v*_p_, AUC, MAX Slope, and Ki-67 (*p* = 0.08, 0.09, 0.07, close to 0.05), and there was no correlation between *v*_e_, TTP, MAX Conc, and Ki-67 (*p* > 0.05) (**Figures 1**, **2**).

**Table 1:** Correlation of the dynamic contrast-enhanced MRI (DCE-MRI) perfusion parameters and Ki-67.

Ki67 and perfusion correlation	Perfusion parameters								
	*K* ^trans^	*k*_ep_	*v*_e_	*v*_p_	TTP	MAX Conc	AUC		MAX Slope
Mean ± SD	0.41 ± 0.2	0.59 ± 0.24	0.74 ± 0.3	0.004 ± 0.008	3.63 ± 1.52	0.85 ± 0.34		5.25 ± 1.17	0.915 ± 0.4
Relevance^a^	0.31	0.30	0.07	0.21	−0.15	0.18		0.21	0.22
*p*-value	0.01	0.01	0.59	0.08	0.22	0.15		0.09	0.07

K^trans^, volume transfer constant; k_ep_, rate constant; v_e_, extravascular extracellular space volume fraction; v_p_, volume ratio; MAX Conc, maximum concentration; TTP, time to peak enhancement; MAX Slope, maximum rising slope; AUC, area under the concentration curve.

a Spearman’s correlation coefficient.

### Correlation of DCE-MRI Perfusion Parameters and CK5/6 and EGFR

Forty-two patients were examined for the immunohistochemical expressions of CK5/6 and EGFR. Among all DCE-MRI perfusion parameters, *v*_p_ was significantly associated exclusively with CK5/6, while the others showed no statistical differences with either CK5/6 or EGFR (**Table 2**).

**Table 2:** Correlation of the dynamic contrast-enhanced MRI (DCE-MRI) perfusion parameters and CK5/6 and epidermal growth factor receptor (EGFR).

	CK5/6			EGFR		
	Negative	Positive	*p*-value	Negative	Positive	*p*-value
*K* ^trans^	0.39 ± 0.21	0.4 ± 0.29	0.68	0.37 ± 0.25	0.42 ± 0.17	0.51
*k*_ep_	0.58 ± 0.27	0.70 ± 0.27	0.39	0.58 ± 0.31	0.60 ± 0.20	0.79
v_e_	0.74 ± 0.33	0.67 ± 0.29	0.68	0.68 ± 0.30	0.79 ± 0.35	0.29
*v*_p_	0.0029 + 0.001	0.016 ± 0.01	0.03*	0.002 ± 0.001	0.006 ± 0.005	0.16
TTP	3.81 ± 1.74	3.84 ± 0.75	0.98	4.12 ± 2.0	3.48 ± 1.14	0.22
MAX Conc	0.82 ± 0.35	0.76 ± 0.33	0.76	0.75 ± 0.37	0.88 ± 0.31	0.23
AUC	5.24 ± 2.38	4.59 ± 1.50	0.6	5.06 ± 2.78	5.30 ± 1.70	0.74
MAX Slope	0.85 ± 0.39	0.96 ± 0.51	0.6	0.78 ± 0.43	0.94 ± 0.33	0.20

K^trans^, volume transfer constant; k_ep_, rate constant; v_e_, extravascular extracellular space volume fraction; v_p_, volume ratio; MAX Conc, maximum concentration; TTP, time to peak enhancement; MAX Slope, maximum rising slope; AUC, area under the concentration curve.

*p < 0.05.

### Correlation of DCE-MRI Perfusion Parameters and Breast Cancer Molecular Subtypes

Among the 67 eligible patients, there were 4 with triple-negative, 17 with HER-2-enriched, 7 with luminal A, and 39 with luminal B breast cancer according to the standard devised by the 12th St Gallen International Breast Cancer Conference (2011).

With regard to the *K* ^trans^ value, HER-2-enriched tumors had statistical differences with luminal A (*p* = 0.03) and luminal B (*p* = 0.04) tumors. The other molecular subtypes did not show any significant differences in terms of *K* ^trans^ (**Table 3**). The *k*_ep_ value of the HER-2-enriched tumors was significantly different from that of luminal A tumors (*p* = 0.03). In terms of the *v*_e_ value, triple-negative tumors had statistical differences with HER-2-enriched tumors (*p* = 0.03), luminal A tumors (*p* = 0.04), and luminal B tumors (*p* = 0.01). A borderline statistical difference was revealed for *v*_p_ between the HER-2-enriched and luminal B lesions (*p* = 0.055). There were statistical differences in TTP between the HER-2-enriched tumors and luminal A (*p* = 0.04) or luminal B (*p* = 0.04) tumors (**Table 3**).

**Table 3:** Correlation of the dynamic contrast-enhanced MRI (DCE-MRI) perfusion parameters and breast cancer molecular subtypes.

	*K* ^trans^	*k*_ep_	*v*_e_	*v*_p_	TTP	MAX Conc	AUC	MAX Slope
Triple negative	0.44 ± 0.77	0.51 ± 0.29	1.12 ± 0.56	0.0016 ± 0.001	4.06 ± 1.74	1.16 ± 0.36	6.77 ± 1.87	0.97 ± 0.23
HER-2 enriched	0.49 ± 0.23	0.67 ± 0.18	0.76 ± 0.33	0.0075 ± 0.006	2.92 ± 0.7	0.93 ± 0.40	5.53 ± 2.25	1.14 ± 0.48
Luminal A	0.31 ± 0.09	0.48 ± 0.17	0.74 ± 0.21	0.0016 ± 0.001	4.31 ± 1.54	0.77 ± 0.08	4.75 ± 1.15	0.79 ± 0.24
Luminal B	0.41 ± 0.21	0.59 ± 0.24	0.69 ± 0.26	0.002 ± 0.0003	3.77 ± 1.69	0.79 ± 0.33	5.07 ± 2.29	0.83 ± 0.38

K^trans^, volume transfer constant; k_ep_, rate constant; v_e_, extravascular extracellular space volume fraction; v_p_, volume ratio; MAX Conc, maximum concentration; TTP, time to peak enhancement; MAX Slope, maximum rising slope; AUC, area under the concentration curve.

When MAX Conc was considered, triple-negative tumors showed a statistical difference with luminal B tumors (*p* = 0.04) and a borderline statistical difference with luminal A tumors (*p* = 0.067), while no significant difference was shown between tumors of other molecular subtypes. There was no significant statistical difference in the AUC values between distinct molecular subtypes. The MAX Slope in HER-2-enriched tumors displayed statistical differences with luminal A (*p* = 0.044) and luminal B (*p* = 0.007) tumors (see **Table 3**).

## Discussion

The tumor size closely pertains to the grade and outcome of breast cancer. González-Sistal et al. demonstrated that larger breast tumors have an inclination for histological grade 3 (10). A significant association between breast cancer grade and tumor size has also been described by Ruibal et al. (11), who found that a larger size was correlated with a higher histological grade. In the present study, the mean tumor size ± SD was 4.48 ± 1.73 cm, which did not correlate with any DCE-MRI perfusion parameters (*p* > 0.05). In addition, Schmitz et al. did not find a definite correlation between the breast tumor dimension and the amount of vascularity adjacent to breast cancer (12). Thus, these results led us to the following speculation: the tumor size made no difference to either the tumor angiogenesis factors of invasive breast carcinoma or to blood perfusion.

Ki-67 is closely correlated with tumor cell proliferation and plays an essential role in tumorigenesis, progression, and metastasis. Besides, Ki-67 exerts a vital role in predicting breast cancer outcomes and evaluating the efficacy of NAC. We found that *K* ^trans^ and *k*_ep_ had a positive association with Ki-67, mainly because the increase in immature blood vessels resulted in the rate of the contrast agent outflow and backflow between the blood vessels and the surrounding tissues speeding up, causing a higher *K* ^trans^ and a higher *k*_ep_ accordingly. Considering the study of Kim et al. based on breast DCE-MRIs, it was shown that *K* ^trans^ was higher in tumors with Ki-67 positivity (0.294 ± 0.293) than that in tumors with Ki-67 negativity (0.132 ± 0.047), with a cutoff value of 5% (5). There was statistical significance between Ki-67 positivity and Ki-67 negativity (*p* = 0.002). Furthermore, *k*_ep_ was higher in tumors with Ki-67 positivity (1.247 ± 1.487) than that in tumors with Ki-67 negativity (0.454 ± 0.310, *p* = 0.005), with a cutoff value of 5%. This was in agreement with our study results.

Kim et al. included IDC, invasive lobular carcinoma, mucinous carcinoma, and other pathological types of breast cancer (5), whereas we only investigated IDC. The results were consistent, and we speculate that DCE-MRI can be used for evaluating the Ki-67 status in common types of breast cancer.

In our study, it was indicated that *v*_p_, AUC, and MAX Slope had borderline correlations with Ki-67 (*p* = 0.08 and close to 0.05). A high *v*_p_ denotes an increase in plasma or blood vessel volume and tumor angiogenesis, elucidating its capability to reflect tumor cell proliferation. The quantitative parameter *v*_e_ and some semiquantitative parameters such as TTP and MAX Conc showed no relationship with Ki-67 (*p* > 0.05), while some semiquantitative parameters such as AUC and MAX Slope showed positive boundary correlations with Ki67. In a previous research by Kim et al., there was no significant correlation between *v*_e_ and Ki-67 (5). Lee et al. reported that the time–intensity curves showed an exclusive correlation with MAX Slope, among the semiquantitative parameters, and Ki-67 expression (13). We believe that many factors could influence the semiquantitative perfusion parameters and that the correlation of Ki-67 with the semiquantitative parameters was inferior to that with the quantitative parameters. Therefore, quantitative perfusion parameters were recommended as indicators for evaluating Ki-67.

CK5/6, confirmed as the distinct cytokeratin that breast basal cells express, is related to the invasion, metastasis, and prognosis of breast cancer (14). In our study of 42 patients, the expression status of CK5/6 was significantly associated exclusively with *v*_p_. There was no statistical significance between the other perfusion parameters and the expressions of CK5/6 and EGFR. The *v*_p_ value was higher in tumors with CK5/6 positivity than that in tumors with CK5/6 negativity, suggesting that proliferative or aggressive breast cancer is more likely to have a rich blood supply. Although CK5/6 and EGFR are noticeably associated with breast cancer invasiveness and cell proliferation, tumors with CK5/6 and EGFR positivity characteristically have several mitotic figures, scant stromal content, and frequent apoptotic cells and show apparent central necrosis and the stromal lymphocytic response (15), which may lead to most of the changes in the DCE-MRI perfusion parameters being less obvious.

Regarded as highly heterogenous tumors, breast cancers with the same morphology tend to have different molecular genetic characteristics. Consequently, the molecular subtypes of breast cancer can provide useful references for tumor heterogeneity, staging, new therapeutic targets, prognostic assessment, individualized treatment strategy, etc. According to the clinically widely used criteria formulated by the 12th St Gallen International Breast Cancer Conference (2011), breast cancer can be classified into four subtypes: luminal A, luminal B, triple negative, and HER-2 enriched (9).

Our research illustrated that the *K* ^trans^ value was higher in HER-2-enriched lesions than that in luminal A or luminal B lesions (*p* < 0.05). This is probably because breast cancer with HER-2 overexpression has higher pathological grading, higher pathological staging, and stronger infiltration, which induced the increase in tumor angiogenesis and immature blood vessels, ultimately leading to an increase in the *K* ^trans^ value. Van et al. found that tumors that are fast growing and has high grading had high *K* ^trans^ values (16). In particular, Han et al. confirmed that the blood vessels of breast cancer with a higher pathological grade were discernibly increased (17).

Similar to the *K* ^trans^ value, *k*_ep_ was higher in HER-2-enriched tumors than that in luminal A tumors, for the same reason. Furthermore, the luminal A subtype showed no HER-2 expression. This was consistent with a finding described by Kim et al., that the *k*_ep_ value was higher in breast tumors with HER-2 positivity (1.065) than that in tumors with HER-2 negativity (0.593) (5). Koo et al. also proved that the mean *k*_ep_ was higher in tumors with a high histologic grade than that in tumors with a low histological grade (18).

In the present study, the *v*_e_ value was more prominent in triple-negative tumors than in HER-2-enriched, luminal A, and luminal B tumors. However, differing from our findings, Li et al. found that the mean *v*_e_ in tumors of the triple-negative subtype was lower than that in other subtypes (19). Triple-negative breast cancers with rich expressions of CK5/6 and EGFR have insufficient stromal content, frequent apoptotic cells, and apparent central necrosis (13). Likewise, all the patients enrolled into our study were diagnosed as breast IDC with a high level of malignancy, which was also the cause of the increased *v*_e_ of triple-negative breast cancer. However, the study of Koo et al. discovered that the *v*_e_ was higher in tumors of the triple-negative subtype than that in tumors of the luminal subtype, which supported our research finding (18).

In a similar mechanism to *K* ^trans^ and *k*_ep_, *v*_p_ was higher in HER-2-enriched tumors than that in luminal B tumors (*p* = 0.055), with a borderline statistical difference, and TTP was faster in HER-2-enriched tumors than that in luminal A and B tumors (*p* < 0.05). Research on the semiquantitative perfusion parameters performed by Kim et al. depicted that TTP had no correlation with HER-2 positivity and HER-2 negativity, which may result from various influencing factors on the semiquantitative perfusion parameters (5).

Necrosis and apoptosis lesions are common in triple-negative breast cancer. Nonetheless, as a subtype with rapid invasion and growth, triple-negative breast cancer has a significantly increased blood supply. Previous research by foreign scholars employing semiquantitative perfusion parameters has reported that the *E*_peak_ was higher in ER or HER-2 positivity than that in ER or HER-2 negativity (5), which was consistent with our study.

The MAX Slope value of HER-2-enriched tumors was higher than that of luminal A or B tumors. When the MAX Slope value of breast IDC increases, the tumor shows tendencies of abundant and immature blood supply. Hence, it can be considered as a definite manifestation of the high malignancy level, the same as the fast decline in the dynamic enhancement time–density curve.

Some limitations of our study should be addressed. Firstly, the number of patients included was relatively small. Further research with a large sample size would be undertaken. Besides, in this study, the AIF was calculated based on the population average method, providing a more unified index and model for the clinic. However, as for an individual, the calculation result would be more reliable if the direct blood supply artery of breast cancer was selected as the source of AIF. Nevertheless, in daily clinical practice, it is an uphill struggle to determine the direct blood supply artery precisely, so the population average method is more preferred. With the development of new technology in the future, research methods would be further improved.

In conclusion, as a quantitative perfusion methodology, DCE-MRI is significantly associated with the biological behaviors and biomarkers of breast cancer. It can serve as a complementary and noninvasive tool to assess and predict the invasion, recurrence, metastasis, and prognosis of breast cancer and may aid in directing clinicians to determine appropriate personalized therapy.

## Data Availability Statement

The raw data supporting the conclusions of this article will be made available by the authors, without undue reservation.

## Ethics Statement

The studies involving human participants were reviewed and approved by Fudan University Shanghai Cancer Center. The patients/participants provided written informed consent to participate in this study.

## Author Contributions

LL reviewed and edited the paper and helped with the methodology. NM wrote the original draft. BY curated the data. WP did the investigation. All authors contributed to the article and approved the submitted version.

## Funding

This project was supported by the Shanghai Municipal Science and Technology Major Project (no. 2018SHZDZX01) and ZJ Lab, Shanghai Center for Brain-Inspired Technology, Clinical Research Plan of SHDC (no. SHDC2020CR4069).

## Conflict of Interest

The authors declare that the research was conducted in the absence of any commercial or financial relationships that could be construed as a potential conflict of interest.

## Publisher’s Note

All claims expressed in this article are solely those of the authors and do not necessarily represent those of their affiliated organizations, or those of the publisher, the editors and the reviewers. Any product that may be evaluated in this article, or claim that may be made by its manufacturer, is not guaranteed or endorsed by the publisher.
